# Does Focus Improve Performance in Elective Surgery? A Study of Obesity Surgery in Sweden

**DOI:** 10.3390/ijerph17186682

**Published:** 2020-09-14

**Authors:** Anna Svarts, Luca Urciuoli, Anders Thorell, Mats Engwall

**Affiliations:** 1 Department of Industrial Economics and Management, KTH Royal Institute of Technology, 10044 Stockholm, Sweden; luca.urciuoli@indek.kth.se (L.U.); mats.engwall@indek.kth.se (M.E.); 2 Zaragoza Logistics Center, MIT International Logistics Program, 50018 Zaragoza, Spain; 3 Karolinska Institutet, Department of Clinical Sciences, Danderyd Hospital, 18288 Stockholm, Sweden; anders.thorell@erstadiakoni.se; 4 Department of Surgery, Ersta Hospital, 11691 Stockholm, Sweden

**Keywords:** focus, quality, productivity, hospitals, bariatric surgery

## Abstract

Recent studies have found positive effects from hospital focus on both quality and cost. Some studies indicate that certain patient segments benefit from focus, while others have worse outcomes in focused hospital departments. The aim of this study was to establish the relationship between hospital focus and performance in elective surgery. We studied obesity surgery procedures performed in Sweden in 2016 (5152 patients), using data from the Scandinavian Obesity Surgery Registry (SOReg) complemented by a survey of all clinics that performed obesity surgery. We examined focus at two levels of the organization: hospital level and department level. We hypothesized that higher proportions of obesity surgery patients in the hospital, and higher proportions of obesity surgery procedures in the department, would be associated with better performance. These hypotheses were tested using multilevel regression analysis, while controlling for patient characteristics and procedural volume. We found that focus was associated with improved outcomes in terms of reduced complications and shorter procedure times. These positive relationships were present at both hospital and department level, but the effect was larger at the department level. The findings imply that focus is a viable strategy to improve quality and reduce costs for patients undergoing elective surgery. For these patients, general hospitals should consider implementing organizationally separate units for patients undergoing elective surgery.

## 1. Introduction

Around 30 years ago, Herzlinger [1,2] was the first to suggest that replacing traditional general hospitals with specialized hospitals emulating focused factories [3] would improve quality and productivity in healthcare services. Since that time, there has been a strong emphasis on the general advantages of focus in healthcare delivery. Research has shown that focused units, which are specialized in a specific segment of patients or offering a narrow range of services, benefit from lower variability and fewer conflicting operational activities and consequently produce healthcare at a lower cost [4], with lower mortality [5,6], and with a shorter length of stay [6] than non-focused units. In practice, the number of specialized healthcare units, such as single specialty hospitals [7,8,9] and ambulatory surgery centres [10], has grown rapidly. Several empirical studies have also shown that specialized units outperform general hospitals both in cost efficiency [8,11] and in patient satisfaction [12,13].

Studies have demonstrated that the positive effect of focus may differ depending on the organizational level considered. In 2008, in a study of clinical trials, Huckman and Zinner [14] discovered that there are benefits of focus not only at an aggregated organizational level (e.g., a hospital) but also at the department level. They concluded that their findings from the context of clinical trials align with the proposal by Skinner [3] in manufacturing management, i.e., to structure industrial manufacturing facilities into focused divisions (‘plants-within-a-plant’) with adequate delineation organizationally as well as physically. This allows for concentration on a single task, without mixing production objectives in terms of quality and volume, and increasing clarity and focus on training, processes, and equipment [14].

Following similar research propositions, but applied in the context of healthcare services, KC and Terwiesch [6] undertook a study aiming to unravel the differences in focus outcomes between three organizational levels: hospital, department, and process. After controlling for selective patient admissions (or ‘cherry-picking’ admission practices), the authors found that hospital-level focus did not bring significant improvements in performance, which was measured as length of stay or mortality rate [6].

However, recent research challenges the generalization of the positive relation between focus and outcomes. Kuntz et al. [15] suggest that the benefits of focus and volume differ between patient segments and that the focus–outcome relationship needs to be determined at a more granular level. The authors distinguish between what they refer to as ‘routine and ‘complex’ patients, placing patients on a complexity spectrum that ranges from elective admissions of patients without comorbidities at one end to emergency admissions of frail patients with multiple comorbidities at the other end. Based on an analysis of in-hospital mortality in 60 German hospitals across 39 disease segments, they argue that for routine patients, focus is the main driver of service quality, while for complex patients, focus does not affect quality. Hence, they suggest that general hospitals should be reorganized as multi-specialty hubs for emergency and non-routine elective services, which are complemented by organizationally separate ‘focused factories’ for routine and elective services (similar hospital operational models are advocated by, e.g., Christensen et al. [16] and Hopp and Lovejoy [17]). The authors call for more research to verify their findings in the context of specific conditions, using condition-specific quality metrics, to be able to give more specific advice to hospital managers who wish to implement such reorganizations. For complex patients, this has been addressed by Miedaner and Sülz [18] in a study of neonatal intensive care units, which confirmed that focus has no effect, or a negative effect, on length of stay in this context. The effect of focus has been extensively evaluated in the context of cardiac care, e.g., [4,5,6,19], but there are few studies in the context of other conditions, especially at the routine end of the complexity spectrum. Routine patients represent a large share of all patients. Comparing elective and emergency surgery, where emergency patients are more complex [15], Prin et al. [20] show that in developed countries, less than one emergency surgery is performed for every ten elective surgeries. In addition, timely and effective elective surgery might reduce the need for emergency services [20]. Therefore, an efficient operational model for treating routine patients is central to curbing healthcare costs while delivering high-quality care.

The purpose of this study is to establish the relationship between focus and performance for [17] patients undergoing obesity surgery. Obesity surgery, or bariatric surgery, is usually elective, and although technically advanced and associated with a risk of complications, it is a routine intervention where standardized protocols exist [21]. Therefore, a study of bariatric surgery may be representative of other routine surgical procedures. Furthermore, organizing bariatric surgery in an efficient way is important from a managerial perspective, since it represents a common elective surgical procedure or, in US hospitals, even the most common elective surgical procedure [22].

Using data from the Scandinavian Obesity Surgery Registry, complemented by a survey distributed to hospitals, this paper provides evidence for a strong association between hospital focus and performance in the context of bariatric surgery. We use a more comprehensive set of outcomes to measure process performance than previous studies of hospital focus. Our study adds to the existing literature on the link between focus and performance by supporting the notion that hospital focus is an important driver of quality and productivity for routine patients. Our findings also inform hospital managers in showing that bariatric surgery benefits from allocation to specialty hospitals or to organizationally separate hospital-within-hospitals focusing on this patient segment.

The rest of the paper is structured as follows. Section 2 presents a brief theoretical background to the hypotheses investigated in our study. Section 3 introduces the methods used, including data, variables, and analyses. Section 4 describes the results. In Section 5, we discuss the results and outline the contributions and limitations of our study. Section 6 presents conclusions and implications for practice.

## 2. Theory

The idea of focus in operations goes back to Skinner’s seminal paper applied to the manufacturing setting stating that the “focused factory will out-produce, undersell, and quickly gain competitive edge over the complex factory”, p. 116 [3]. Benefits are expected from repetition, concentration, and less distraction from conflicting activities due to lower volume outside the focal segment. The idea of focus benefits has also been transferred to the healthcare context [1,2,4,5,6]. As an example, in business schools, the concept of focused factories has long been taught using a case study from the healthcare sector, Shouldice Hospital, which successfully specializes in hernia repair procedures [23].

### 2.1. Hospital Focus

Many recent studies have found positive effects from hospital focus on both quality and cost [4,5,6,24,25].

Recent studies have suggested that even if focus benefits certain patient segments, the general effect of focus is moderated by patient complexity [15,26]. Their findings suggest that while “routine patients” [15] with well-defined health problems benefit from focus, complex patients requiring care from multiple medical specialties, and with high degrees of uncertainty and unpredictability related to their medical conditions, benefit from a broader operational scope [5]. For instance, research has indicated that focused health units are unsuitable for obstetric care due to the difficulty of separating “simple” conditions from complex conditions in the context of pregnancy [27]. As mentioned above, there has similarly been no positive effect from focus identified in the complex setting of neonatal intensive care [18].

Thus, it is suggested that the effect of hospital focus is contingent on the health issues of the patients [15], following the classical advice that the appropriate organizational design is contingent on the nature of the operations [28,29,30]. The new “contingency theory of hospital focus” [15] predicts that focus will have a positive effect on outcomes for routine patients. In the context of obesity surgery—which is elective and involves procedures that, although technically advanced and associated with a risk of complications, are standardized and have a relatively low degree of uncertainty—this theory would predict that hospital focus is positively associated with outcomes.

As the reviewed studies have not reached a consolidated position on the relationship between hospital focus and outcomes, we suggest the following hypothesis:

**Hypothesis 1 (H1).**  An increase in hospital focus on obesity surgery is associated with improved outcomes for patients undergoing obesity surgery.

### 2.2. Department Focus

In a study of the impact of focus on productivity in clinical trials, Huckman and Zinner [14] distinguish the effects of focus at the firm level from the effects of focus at the divisional level. Using Skinner’s [3] concept of a plant-within-a-plant, they found that a division may gain benefits from being focused even when the overall organization is unfocused. This was further developed by KC and Terwiesch [6] who in the context of cardiac care examined the effects of focus at three levels of the organization: hospital, department, and process. We follow these authors and study the impact of focus at both the hospital level and department levels.

In line with the reasoning of Huckman and Zinner [14], as well as findings from previous studies [6,24,25], we hypothesize a positive association between focus and outcome at the department level as well. The operational benefits of focus, through mechanisms such as specialized technical equipment and the adaptation of organization and processes to the focal segment, can be assumed to be more pronounced at the department level compared to the hospital level. This would predict higher benefits of focus at the department level than at the hospital level.

As the previous studies provided limited findings on the relationship between department focus and outcomes and the relationship between department-level and hospital-level focus, we suggest the following hypotheses:

**Hypothesis 2 (H2).**  An increase in department focus on obesity surgery is associated with improved outcomes for patients undergoing obesity surgery.

**Hypothesis 3 (H3).**  The benefits of focus are higher at the department level than at the hospital level.

## 3. Methods

### 3.1. Data and Sample

Data on obesity surgeries in Sweden were retrieved from the Scandinavian Obesity Surgery Registry (SOReg) and a survey collecting complementary data from the hospitals that report to SOReg.

The SOReg database is a national quality registry constituting one of the most comprehensive databases of bariatric surgery in the world. The registry is continuously audited with less than 2% incorrect data entries [31]. It contains national data from over 99% of the bariatric surgeries in Sweden since 2007 [31], including those performed with (1) gastric bypass and (2) sleeve gastrectomy. The former includes rerouting nutrients to bypass the stomach, duodenum, and the proximal small intestine in order to reduce the sense of appetite. The latter involves the resection of a major part of the stomach, with the same of intent of reducing appetite and increasing the sensation of satiation. The registry compiles data on patient characteristics, surgical details, and outcomes including complications. Nineteen variables are mandatory for all patients and are recorded at the time of surgery and at several follow-up visits after surgery. Other variables are sometimes mandatory, depending on the type of surgical procedure. Additional optional variables, including quality-of-life-surveys completed by patients before and after surgery, are used in some hospitals. By the end of December 2016, the database included data on 58,417 patients. For this study, we collected data for all primary gastric bypass and sleeve gastrectomies performed between 1st January and 31st December 2016. Excluding repeat surgeries (*n* = 197), we identified a total of 5152 adult patients who underwent surgery in 2016 and patients who had other surgical procedures performed at the same time as their bariatric surgery (*n* = 265).

The SOReg registry contains comprehensive data on all obesity surgery patients, but in order to measure focus, we also needed information about other patients at the hospitals who were not covered by the registry. Hence, in order to measure the degree of focus in obesity surgery, we collected complementary data through a short survey sent to all Swedish hospitals reporting to SOReg. The survey consisted of a questionnaire asking for the total number of admissions at the hospital as well as the total number of surgical procedures at the department where obesity surgery was performed between 1st January and 31stDecember 2016. The questionnaire was sent by email to the SOReg coordinator at each hospital in March 2019 and was followed by reminders via telephone when needed. Of the 44 hospitals that performed obesity surgery in 2016, 39 responded to the survey, corresponding to a response rate of 89%. The non-respondents included two clinics that had been closed down. Two of the remaining hospitals were reached by phone and attempted to respond, but they could not deliver the historical aggregated data that was necessary for this study due to organizational and software changes. Only one of the hospitals did not respond, despite repeated reminders. There was no reason to assume a systematic bias in the missing responses.

The study was approved by the Regional Ethical Review Board in Stockholm, Sweden (registration number 2018/695-31/3).

### 3.2. Variables

We used a comprehensive set of outcomes as performance indicators: *complications* at three points in time, *procedure time*, and *length of stay*. Separate regressions were run for each of the five outcome variables.

*Complications* are considered the most reliable measure of quality in bariatric surgery, and they are preferable to other measures such as re-operations, which are too rare to be a reliable measure for low-volume hospitals, or mortality, which is too rare to be a reliable measure regardless of hospital volume [32]. The data were filtered to include all complications that required surgical intervention under general anesthesia or intensive care (complication grade > 3a according to the Clavien–Dindo classification [33,34] as binary variables at three points of time: (1) during surgery (intraoperative complications); (2) within 30 days after surgery as reported at the follow-up visit approximately six weeks after surgery (30-day complications); and (3) occurring between 30 days and one year after surgery, as reported at the follow-up visit approximately one year after surgery (one-year complications).

*Procedure time* and *length of stay* are indicators of both quality and cost in bariatric surgery. Procedure time, measured as operating time in minutes (time from first skin incision to completion of wound closure), is an established quality indicator, since shorter procedure times have been shown to be correlated with lower mortality [35], fewer complications [35,36], and shorter length of stay [36]. Shorter procedure times can also be a driver of lower cost per procedure, since they allow more procedures to be performed using the same operating facilities and staff. Similarly, *length of stay*, measured as days from admission to discharge, is a widely used indicator of quality in bariatric surgery (see, e.g., the outcome measures included in the systematic reviews of Markar et al. [37] and Zevin et al. [38]). Longer postoperative hospital stay is also a cost driver, since this consumes more medical and physical resources and consequently increases the cost per patient.

*Hospitalfocus* and *department focus* constituted the two explanatory variables. In line with previous studies [4,5,6,18], we conceptualize focus as an emphasis on a particular service segment (i.e., obesity surgery) and operationalize it as the number of obesity surgery patients in relation to the total number of patients. The annual number of obesity surgery admissions divided by the annual number of total admissions to the hospital was used as our measure of *hospital focus*. The annual number of obesity surgery procedures divided by the annual number of all surgical procedures was used as our measure of *department focus*.

Our sample included many low-focus hospitals and departments and relatively few high-focus units. To address the resulting positive skew of the distributions, hospital focus and department focus were normalized using the natural log of the variables [39].

The following control variables were included as covariates to control for case mix: age; sex; pre-surgery BMI; and the comorbidities sleep apnea, hypertension, diabetes, and dyslipidemia. Type of procedure (gastric bypass or sleeve gastrectomy) was included as an additional covariate. We also controlled for procedural volume, measured as the number of primary bariatric surgical procedures performed in 2016 at the hospital where the surgery was performed.

### 3.3. Analyses

Multilevel regression analyses using complete case analysis were conducted using Stata version 14.1, with individuals nested within clinic-level random effects. Adopting a multilevel model structure has become increasingly common when comparing the performance of multiple healthcare providers [40,41] and are appropriate when observations are not independent of each other due to shared group characteristics. Multilevel regression models have been used in previous studies of focus in hospitals e.g., [4,18]. The multilevel modelling approach, as applied to patients in hospitals, is based on the assumption that there are hospital-specific random effects that are drawn from a common distribution. In our case, the predictor variables were measured at the hospital level, while the output variables were measured at the individual level, and this structure necessitated a multilevel modelling approach with random intercepts that allow different performance baselines for each hospital [42].

Three regression models were developed and tested: the first using multilevel logistic regression, the second using multilevel linear regression, and the last using multilevel Poisson regression (following the guidelines by Rabe-Hesketh and Skrondal [42,43]). Logistic regression was used to assess complications, as this variable is binary. Linear regression was used to assess procedure time, as this is a continuous variable. Poisson regression was used to assess length of stay, as this is measured in whole days, i.e., discrete data with non-negative integer values. All regressions used the maximum likelihood (ML) estimation method (see, e.g., [4]).

Furthermore, we employed a complete case analysis, using only observations with complete data in each regression [39]. For most variables (with a few exceptions discussed below), less than 0.2% of the values were missing. The focus variables were missing for 8.9% of patients due to the survey response rate, as described in the section “Data and sample” above. Data on 30-day complications were missing for 3.9% of patients, reflecting a small number of individuals that either did not show up for the six-week follow-up visit or had a follow-up visit at another clinic that did not report to the registry. For one-year complications, 14% of the values were missing, presumably reflecting patients that did not show up for the one-year follow-up visit. Patients with serious complications could be assumed to have returned to the hospital; consequently, patients with a missing value for one-year complications are likely to belong to the great majority of patients without major complications.

## 4. Results

### 4.1. Descriptive Statistics

Table 1 describes the patients included in the study (*n* = 5152). Seventy-seven percent of the patients were female, and the average age was 41 years. The average BMI before surgery was 41 kg/m^2^. The rate of complications was around 2% at all time periods measured: during surgery, within 30 days, and between 30 days and one year after surgery. Procedure time ranged from 14 to 250 min, with an average of 56.89 min, and the mean length of stay was 1.47 days, with a maximum stay of 50 days.

Table 2 shows descriptive statistics for the hospitals where the surgeries were performed (*n* = 39). Hospital annual volume ranged from less than 250 inpatient admissions to over 90,000 admissions, with a mean of 18,436 admissions. The departments performing obesity surgery ranged from departments with fewer than a total of 100 inpatient surgeries per year to a high-volume department with more than 6000 inpatient surgeries, with an average annual volume of 2034 surgeries. The annual number of obesity surgery procedures performed at the hospital ranged from 4 to 591 with a mean of 121 procedures. The average focus on obesity surgery at the hospital level was 0.076, and at the department level, it was 0.12, reflecting the fraction of obesity surgery patients and obesity surgery procedures, respectively. The sample included both hospitals with an exclusive focus on obesity surgery and those where obesity surgery represented a very small share of the total caseload: i.e., focus values range from 0.94 to 0.00 at both the hospital and the department level (see Table 2).

### 4.2. Effects of Hospital-Level and Department-Level Focus

The results of the multilevel regression analyses are displayed in Table 3, Table 4 and Table 5, which list our main variables in the first panel, report variance components of the mixed model in the second panel, and provide basic model statistics in the bottom panel. Columns with hospital focus models are marked as (1) and columns with department focus models are marked as (2). The model specifications include all the patient-level variables listed in Table 1. However, to simplify the exposition of our results, we do not show all the control variables in the tables. Tables with the full regression results including all control variable coefficients are provided in Appendix A. For the models with intraclass correlation (ICC) estimates, the ICC indicates substantial variation at the group level (hospitals), which supports our choice of multilevel modeling. Since Stata does not estimate the ICC for Poisson regressions, these are missing in Table 5, but the significant random intercepts still support the multilevel specification.

Our first hypothesis predicted that an increase in hospital focus would result in improved outcomes for patients undergoing obesity surgery. Hypothesis 1 is supported for three of the five outcome measures: 30-day complications (*p* = 0.015, β = −0.195), 1-year complications (*p* = 0.006, β = −0.211), and procedure time (*p* = 0.005, β = −3.144). Since the focus variables were log transformed, the regression coefficients relate to a change of one logit unit in the explanatory variable. However, the effect size in relation to the original scale of hospital focus and department focus could be readily calculated; see, e.g., [44]. For interpretation of the linear regressions, the effect of a certain percentage change in the focus variable is given by multiplying the regression coefficient by the natural log of the change factor: i.e., β × ln(1 + x × 0.01). For the logistic and Poisson regressions, the odds ratio, and the incidence rate ratio, respectively, the effect of an x percent change in the focus variable is given by exponentiating the change factor by the regression coefficient: i.e., (1 + x × 0.01)^β^. A 10% increase in hospital focus reduces the risk of 30-day complications by 1.8% (*p* < 0.05), reduces the risk of 1-year complications by 2.0% (*p* < 0.01), and decreases the mean procedure time by 3.0 min (*p* < 0.01). There is no statistically significant effect of hospital focus on intraoperative complications or on length of stay.

Our second hypothesis predicted that an increase in department focus would result in improved outcomes for patients undergoing obesity surgery. Hypothesis 2 is supported by the same three outcome measures as the first hypothesis: i.e., 30-day complications (*p* = 0.000, β = −0.384), 1-year complications (*p* = 0.049, β = −0.270), and procedure time (*p* = 0.003, β = −5.981). When relating the effect size to the original focus scale, a 10% increase in department focus reduces the risk of 30-day complications by 3.6% (*p* < 0.01), reduces the risk of 1-year complications by 2.5% (*p* < 0.05), and decreases the mean procedure time by 5.7 min (*p* < 0.01). Similarly as for hospital focus, there was no statistically significant effect from department focus on intraoperative complications or length of stay.

Our third hypothesis predicted that the benefits of focus are higher at the department level than at the hospital level. Hypothesis 3 is supported by all outcome measures. As seen already, all three outcome measures that are significantly affected by focus at both the hospital level and the department level have a larger effect size for department-level focus. The difference is smallest for 1-year complications (hospital level β = −0.211, and department level β = −0.270), where also the statistical significance is also strongest at the hospital level (*p* < 0.01). We find the same pattern in the regression coefficients for intraoperative complications and length of stay, with a larger magnitude of effect from focus at the department level (although this is not statistically significant).

## 5. Discussion

The present study reveals a positive effect of hospital focus and department focus on performance. Significant effects were found on the number of complications within 30 days and one year after surgery, as well as on procedure time, after controlling for the effects of different patient risk factors. The effect size was relatively small, with a 10% increase in focus corresponding to 2–4% fewer complications and a reduction in procedure time of 3–6 min. For obesity surgery, where the overall risk of complications is low, this may not be of great clinical significance. However, the relationship established in this study could have a far greater clinical significance for other types of elective surgeries with higher overall complication rates: e.g., certain cancer surgeries.

We found no statistically significant relationship between focus and length of stay. For length of stay, there are large inter-hospital differences that may be explained by different operational characteristics—or even traditions—not related to focus, such as local discharge criteria and geographical distance between the hospital and patients’ homes. We note that also previous studies show mixed results on the effects of focus on length of stay: Capkun, Messner, and Rissbacher [24] reported that focus decreases the length of stay for hospitals overall, while KC and Terwiesch [6] did not find a statistically significant association between focus at any organizational level and length of stay in the context of cardiac care.

Thus, the present study shows that hospital units with higher focus have better outcomes. There are two possible explanations of this relationship: focus might drive performance or performance might drive focus. In the former case, focus enables learning, specialization, and higher quality in healthcare procedures, which results in better outcomes. In the latter case, good outcome records result in stronger demand for obesity surgery in relation to other healthcare services, which, in the long run, makes the hospital more focused. In Sweden, the individual choice of healthcare provider is limited, and the majority of obesity surgery patients whose procedures are tax-funded are typically remitted to the closest provider. Given this empirical context, it is plausible to assume that it is focus that drives performance in this setting. However, the dynamics of causation might differ between different contexts and healthcare systems, which calls for these contextual factors to be taken into account in further research.

Our results are not consistent with the recent study by Miedaner and Sülz [18], which found negative effects from focus in neonatal intensive care. A possible explanation of the incongruent results would be that patient characteristics moderate the impact of focus on performance. In that case, the different characteristics of obesity surgery patients and patients in neonatal care could explain the mixed findings. According to a recently presented contingency theory of hospital focus [15], the differences in results may be explained by the fact that patients in the two studies are widely distributed on the scale between highly complex, unpredictable, emergency care, and highly predictable, routine, elective care. It would be unreasonable to assume that obesity surgery is on the extreme routine end of this scale, given that many obese patients have comorbidities that increase unpredictability and that many simpler elective procedures exist. However, we argue that the current standardized nature of the obesity surgery procedures means that patients undergoing obesity surgery can be characterized as relatively routine elective patients, and that this is a likely explanation of our results in relation to the results of Miedaner and Sülz [18].

We also found that the benefits of focus are stronger at the more granular organizational level. This hypothesis was supported by all five outcome measures. Our results are consistent with the findings of KC and Terwiesch [6] in the context of cardiac care, in which length of stay and mortality were reduced more by department-level (operating unit) and process-level focus than by hospital-level (firm) focus.

The present study adds to the emerging contingency theory of hospital focus by supporting the notion that hospital focus is an important driver of quality and productivity for patients located toward the routine end of the complexity spectrum [15]. A key strength of this study is that it uses a more comprehensive set of relevant outcomes to measure process performance than previous studies looking at the link between focus and performance.

This study has some limitations. It uses data from the Scandinavian Obesity Surgery Registry, and this may limit the generalizability to other countries—in particular to developing countries—where conditions for quality and cost in healthcare are substantially different from those in Scandinavia. However, the study benefits from using one of the most comprehensive databases on obesity surgery available, and it is plausible that our results are valid for countries in North America and Europe with similar conditions as those found in Sweden. Our study is also limited to studying only one type of surgery; therefore, its generalizability for other surgical procedures could be questioned. Further studies could also include the effects of focus on the hospital as a whole or the spillover effects [5] of the hospital’s focus on related patient segments, as well as further control variables, accounting for, e.g., seasonal and day-of-week effects. Moreover, since this study was limited to quality outcomes and productivity measures (procedure time and length of stay), studies that measure cost performance more explicitly are also called for.

## 6. Conclusions and Implications

This study addresses the relationship between focus and performance for patients undergoing elective surgery. Based on a study of obesity surgery, we conclude that focus has a positive effect on elective surgery performance at both the hospital level and the department level. Significant effects were found on the number of complications within 30 days and one year after surgery, as well as on procedure time after controlling for the effects of different patient risk factors.

This study has several implications for how obesity surgery should be organized to improve efficiency and patient safety. Firstly, the establishment of specialty hospitals and other types of specialized healthcare providers should be encouraged. Focus leads to improved performance at the hospital level, which could be explained by a lower level of distraction from other processes and patient segments. Secondly, general hospitals can emphasize particular areas in order to increase hospital focus. It may be appropriate to emphasize a particular service area in an operational way and thus achieve a higher proportion of these patients, even when a broad range of services is required from the hospital [4]. Thirdly—and probably most feasible to implement—general hospitals should create hospitals-within-hospitals with a high level of department focus. Our study shows that for patients undergoing obesity surgery, a focused department is more important than a focused hospital, and focus is more important than procedural volume. These hospital-within-hospital departments can be complemented by flexible multi-specialty services that meet the needs of complex patients [15,16,17]. An organizational redesign to create a hospital-within-a-hospital can be guided by case studies of successful implementations of focused hospital units e.g., [25,45].

Our findings clearly support the use of these three guidelines for organizing obesity surgery. They also indicate that similar organizational designs are appropriate for other elective surgeries that involve standardized procedures. However, more research is needed to provide advice for other specific medical conditions.

## Figures and Tables

**Table 1:** Descriptive statistics for bariatric surgery patients in Sweden 2016.

Characteristic	*n* (%)	Mean	Std. Dev.	Median	Min.	Max.
Age		40.76	11.33	41.00	18	74
Female	3983 (77)					
Male	1169 (23)					
Gastric bypass	3278 (64)					
Gastric sleeve	1838 (36)					
Pre-surgery BMI		41.03	5.66	40.40	28.65	78.60
Sleep apnea	475 (9.2)					
Hypertension	1214 (24)					
Diabetes	603 ((12)					
Dyslipidemia	482 (9.4)					
Intraoperative complications	111 (2.2)					
30-day complications	126 (2.5)					
One-year complications	101 (2.3)					
Procedure time		56.89	24.78	53	14	250
Length of stay		1.47	1.73	1	0	50

Patients (*n* = 5152).

**Table 2:** Descriptive statistics for hospitals performing bariatric surgery in Sweden in 2016.

Characteristic	Mean	Std. Dev.	Median	Min.	Max.
Admissions	18,436	19,303	12,089	241	90,837
Surgical procedures (in-patient)	2034	1462	1845	98	6268
Obesity surgery procedures	121	123.85	86	4	591
Gastric bypass	76	88.12	44	0	438
Sleeve gastrectomy	45	56.89	27	0	260
Hospital-level focus	0.076	0.207	0.006	0.000	0.943
Department-level focus	0.117	0.181	0.052	0.001	0.943

Hospitals (*n* = 39).

**Table 3:** Multilevel mixed-effect regression of hospital focus on complications.

	Intraoperative Complications		30-Day Complications		One-Year Complications	
	(1)	(2)	(1)	(2)	(1)	(2)
	β (SE)	β (SE)	β (SE)	β (SE)	β (SE)	β (SE)
Log hospital focus	−0.0657		−0.195 **		−0.211 ***	
	(0.103)		(0.0799)		(0.0773)	
Log department focus		−0.114		−0.384 ***		−0.270 **
		(0.150)		(0.103)		(0.137)
Random effects:						
Constant (std. dev.)	0.662 **	0.653 *	0.230	7.56 × 10^−17^	0.182	0.231
	(0.169)	(0.169)	(0.115)	(1.1856 × 10^−16^)	(0.223)	(0.197)
Observations (*n*)	4719	4719	4609	4609	4130	4130
Number of groups	39	39	39	39	39	39
ICC: 95% CI	[0.0468; 0.265]	[0.0448; 0.263]	[0.00229; 0.102]	[3.7356 × 10^−36^; 8.0856 × 10^−31^]	[0.000082; 0.553]	[0.00057; 0.315]

Robust standard errors in parentheses; * *p* < 0.1, ** *p* < 0.05, *** *p* < 0.01. Control variables included procedural volume and the patient level controls age, sex, pre-surgery body mass index (BMI), surgery method, sleep apnea, hypertension, diabetes, and dyslipidemia. (1) Hospital level, (2) Department level.

**Table 4:** Multilevel mixed-effect linear regression of focus on procedure time.

	Procedure Time	
	(1)	(2)
	β (SE)	β (SE)
Log hospital focus	−3.144 ***	
	(1.131)	
Log department focus		−5.981 ***
		(1.992)
Random effects:		
Constant (std. dev.)	12.58 ***	12.08 ***
	(1.849)	(1.643)
Residual (std. dev.)	16.45 ***	16.45 ***
	(1.031)	(1.031)
Observations (*n*)	4719	4719
Number of groups	39	39
ICC: 95% CI	[0.238; 0.524]	[0.232; 0.491]

Robust standard errors in parentheses; * *p* < 0.1, ** *p* < 0.05, *** *p* < 0.01. Control variables included procedural volume and the patient level controls age, sex, pre-surgery BMI, surgery method, sleep apnea, hypertension, diabetes, and dyslipidemia. (1) Hospital level, (2) Department level.

**Table 5:** Multilevel mixed-effect Poisson regression of focus on length of stay.

	Length of Stay	
	(1)	(2)
	β (SE)	β (SE)
Log hospital focus	−0.0188	
	(0.0361)	
Log department focus		−0.0702
		(0.0527)
Random effects:		
Constant (std. dev.)	0.318 ***	0.314 ***
	(0.0314)	(0.0301)
Observations (N)	4714	4714
Number of groups	39	39

Robust standard errors in parentheses; * *p* < 0.1, ** *p* < 0.05, *** *p* < 0.01. Control variables included procedural volume and the patient level controls age, sex, pre-surgery BMI, surgery method, sleep apnea, hypertension, diabetes, and dyslipidemia. (1) Hospital level, (2) Department level.

**Figure A1:**
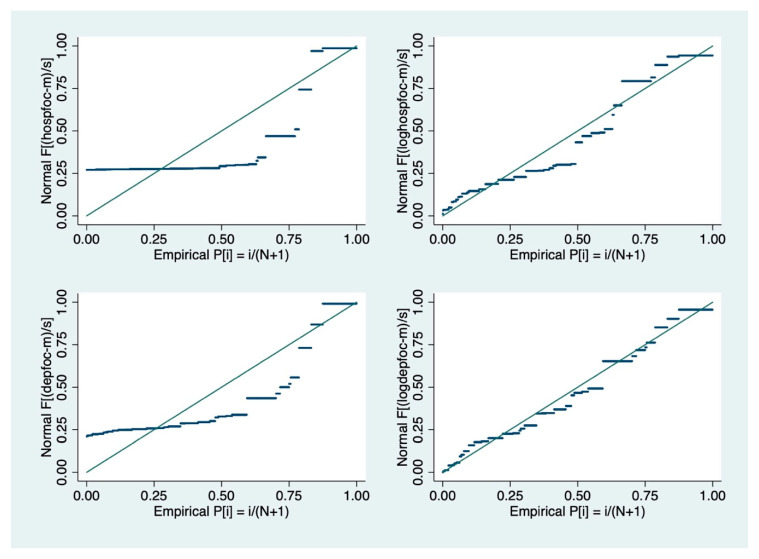
Normal probability plot (standardized) of transformed variables.

## Appendix A. Regression Tables and Model Specifications

For the complication outcomes at three points in time, we use logistic regression models, which were amended to have random effects $u_{j}$ for each hospital *j*. Defining, respectively, $\pi_{ij}=\mathrm{Pr}\left(intraopcomplications_{ij}=1\right)$, $\pi_{ij}=\mathrm{Pr}\left(30daycomplications_{ij}=1\right)$, and $\pi_{ij}=\mathrm{Pr}\left(oneyearcomplications_{ij}=1\right)$, we have:

$$logit\left(\pi_{ij}\right)=\beta_{0}+\beta_{1}loghospfocus_{ij}+\beta_{2}procvol_{ij}+{ℱ}_{ij}+u_{j}$$

and

$$logit\left(\pi_{ij}\right)=\beta_{0}+\beta_{1}logdepfocus_{ij}+\beta_{2}procvol_{ij}+{ℱ}_{ij}+u_{j}$$

for j=1,. . . , 39 hospitals, with $i=1,\;.\;.\;.\;,\;n_{j}$ patients in hospital j, and the following patient-level control variables: ${ℱ}_{ij}=\beta_{3}gbp_{ij}+\beta_{4}sleeve_{ij}+\beta_{5}age_{ij}+\beta_{6}female_{ij}+\beta_{7}bmi_{ij}+\beta_{8}sleepapnea_{ij}+\beta_{9}hypertension_{ij}+\beta_{10}diabetes_{ij}+\beta_{11}dyslipidemia_{ij}$.

**Table A1:** Multilevel mixed-effect regression of hospital focus on complications.

	Intraoperative Complications		30-Day Complications		One-Year Complications	
	(1)	(2)	(1)	(2)	(1)	(2)
	β (SE)	β (SE)	β (SE)	β (SE)	β (SE)	β (SE)
Log hospital focus	−0.0657		−0.195 **		−0.211 ***	
	(0.103)		(0.0799)		(0.0773)	
Log department focus		−0.114		−0.384 ***		−0.270 **
		(0.150)		(0.103)		(0.137)
Procedural volume	−0.00226	−0.00222	0.00155	0.00184 **	0.00163 *	0.00107
	(0.00221)	(0.00222)	(0.000950)	(0.000731)	(0.000886)	(0.000831)
Gastric bypass	0.324	0.314	−0.0502	−0.120	−0.441	−0.480
	(0.481)	(0.473)	(0.900)	(0.987)	(0.803)	(0.788)
Sleeve gastrectomy	0.0886	0.0685	−0.547	−0.631	−1.831 **	−1.918 **
	(0.537)	(0.535)	(0.964)	(1.048)	(0.863)	(0.845)
Age	0.0183	0.0183	0.00635	0.00594	−0.00564	−0.00619
	(0.0129)	(0.0129)	(0.00836)	(0.00839)	(0.00835)	(0.00824)
Female	−0.174	−0.171	−0.0760	−0.0638	0.138	0.148
	(0.239)	(0.240)	(0.225)	(0.226)	(0.315)	(0.316)
Pre-surgery BMI	0.0101	0.0105	−0.0181	−0.0159	−0.0241	−0.0211
	(0.0181)	(0.0183)	(0.0211)	(0.0205)	(0.0290)	(0.0285)
Sleep apnea	0.334	0.335	0.263	0.274	−0.238	−0.211
	(0.223)	(0.224)	(0.266)	(0.267)	(0.390)	(0.390)
Hypertension	0.0288	0.0292	−0.246	−0.246	−0.317	−0.316
	(0.269)	(0.272)	(0.239)	(0.238)	(0.284)	(0.286)
Diabetes	0.186	0.184	0.0645	0.0670	−0.124	−0.114
	(0.303)	(0.299)	(0.381)	(0.382)	(0.361)	(0.364)
Dyslipidemia	0.336	0.336	−0.371	−0.372	−0.0405	−0.0339
	(0.267)	(0.268)	(0.302)	(0.303)	(0.382)	(0.382)
Random effects:						
Constant (std. dev.)	0.662 **	0.653 *	0.230	7.56 × 10^−17^	0.182	0.231
	(0.169)	(0.169)	(0.115)	(1.18 × 10^−16^)	(0.223)	(0.197)
Observations (N)	4719	4719	4609	4609	4130	4130
Number of groups	39	39	39	39	39	39
ICC: 95% CI	[0.0468; 0.265]	[0.0448; 0.263]	[0.00229; 0.102]	[3.73 × 10^−36^; 8.08 × 10^−31^]	[0.000082; 0.553]	[0.00057; 0.315]

Robust standard errors in parentheses; * *p* < 0.1, ** *p* < 0.05, *** *p* < 0.01. (1) Hospital level, (2) Department level.

For the procedure time outcome, we use random-intercept linear regression models:

$$\begin{array}{c} proctime_{ij}=\beta_{0}+\beta_{1}loghospfocus_{ij}+\beta_{2}procvol_{ij}+{ℱ}_{ij}+u_{j}+\epsilon_{ij}\\ proctime_{ij}=\beta_{0}+\beta_{1}logdepfocus_{ij}+\beta_{2}procvol_{ij}+{ℱ}_{ij}+u_{j}+\epsilon_{ij} \end{array}$$

for j, i, and ${ℱ}_{ij}$ as above.

**Table A2:** Multilevel mixed-effect linear regression of focus on procedure time.

	Procedure Time	
	(1)	(2)
	β (SE)	β (SE)
Log hospital focus	−3.144 ***	
	(1.131)	
Log department focus		−5.981 ***
		(1.992)
Procedural volume	−0.0329 **	−0.0249 *
	(0.0159)	(0.0148)
Gastric bypass	−75.55 ***	−75.58 ***
	(11.72)	(11.71)
Sleeve gastrectomy	−90.43 ***	−90.47 ***
	(11.69)	(11.68)
Age	0.131 ***	0.131 ***
	(0.0279)	(0.0279)
Female	−3.987 ***	−3.978 ***
	(0.676)	(0.676)
Pre-surgery BMI	0.351 ***	0.351 ***
	(0.0883)	(0.0884)
Sleep apnea	2.503 **	2.507 **
	(1.123)	(1.124)
Hypertension	−1.263	−1.265
	(0.945)	(0.945)
Diabetes	1.299	1.274
	(0.867)	(0.866)
Dyslipidemia	1.453 *	1.440 *
	(0.749)	(0.749)
Random effects:		
Constant (std. dev.)	12.58 ***	12.08 ***
	(1.849)	(1.643)
Residual (std. dev.)	16.45 ***	16.45 ***
	(1.031)	(1.031)
Observations (N)	4719	4719
Number of groups	39	39
ICC: 95% CI	[0.238; 0.524]	[0.232; 0.491]

Robust standard errors in parentheses; * *p* < 0.1, ** *p* < 0.05, *** *p* < 0.01. (1) Hospital level, (2) Department level.

For the length of stay outcome, we use random-intercept Poisson regression models with a set of random effects $u_{j}$.

$$\log(\mu_{ij})=\beta_{0}+\beta_{1}loghospfocus_{ij}+\beta_{2}procvol_{ij}+{ℱ}_{ij}+u_{j}$$

$$\log(\mu_{ij})=\beta_{0}+\beta_{1}logdepfocus_{ij}+\beta_{2}procvol_{ij}+{ℱ}_{ij}+u_{j}$$

for j, i, and ${ℱ}_{ij}$ as above.

**Table A3:** Multilevel mixed-effect Poisson regression of focus on length of stay.

	Length of Stay	
	(1)	(2)
	β (SE)	β (SE)
Log hospital focus	−0.0188	
	(0.0361)	
Log department focus		−0.0702
		(0.0527)
Procedural volume	−0.000743 *	−0.000474
	(0.000420)	(0.000394)
Gastric bypass	−0.463 **	−0.465 **
	(0.214)	(0.214)
Sleeve gastrectomy	−0.430 **	−0.431 **
	(0.206)	(0.205)
Age	−0.00295 **	−0.00295 **
	(0.00146)	(0.00146)
Female	0.0766 *	0.0769 *
	(0.0406)	(0.0407)
Pre-surgery BMI	0.00330	0.00323
	(0.00240)	(0.00242)
Sleep apnea	0.0468	0.0468
	(0.0739)	(0.0739)
Hypertension	0.0337	0.0334
	(0.0494)	(0.0494)
Diabetes	0.183	0.182
	(0.115)	(0.115)
Dyslipidemia	−0.0235	−0.0244
	(0.0667)	(0.0666)
Random effects:		
Constant (std. dev.)	0.318 ***	0.314 ***
	(0.0314)	(0.0301)
Observations (*N*)	4714	4714
Number of groups	39	39

Robust standard errors in parentheses; * *p* < 0.1, ** *p* < 0.05, *** *p* < 0.01. (1) Hospital level, (2) Department level.

## Appendix B. Variable Transformations

Our sample included many low-focus hospitals and departments. To address the resulting positive skew of the distributions, hospital focus and department focus were normalized using the natural log of the variables. In this appendix, we present the standardized normal probability plot of the original focus variables and the log transformed focus variables. The upper left plot shows hospital focus and the lower left plot shows department focus as originally measured. On the right-hand side are plots of the transformed variables (the upper right plot shows a natural log of hospital focus, and the lower right plot shows a natural log of department focus), which are closer to the ideal line.
